# Matrix Metalloproteinase-9 Mediates RSV Infection *in Vitro* and *in Vivo*

**DOI:** 10.3390/v7082817

**Published:** 2015-07-30

**Authors:** Michele Y.F. Kong, Richard J. Whitley, Ning Peng, Robert Oster, Trenton R. Schoeb, Wayne Sullender, Namasivayam Ambalavanan, John Paul Clancy, Amit Gaggar, J. Edwin Blalock

**Affiliations:** 1 Departments of Pediatrics, University of Alabama at Birmingham, PPS 102, 1600 5th Ave South, Birmingham, AL 35233, USA; E-Mails: rwhitley@peds.uab.edu (R.J.W.); npeng@peds.uab.edu (N.P.); nambalavanan@peds.uab.edu (N.A.); 2 Departments of Medicine, University of Alabama at Birmingham, PPS 102, 1600 5th Ave South, Birmingham, AL 35233, USA; E-Mails: roster@uabmc.edu (R.O.); agaggar1@uab.edu (A.G.); blalock@uab.edu (J.E.B.); 3 Departments of Genetics, University of Alabama at Birmingham, PPS 102, 1600 5th Ave South, Birmingham, AL 35233, USA; E-Mail: trs@uab.edu; 4 Center for Global Health, Colorado School of Public Health, 13199 E Montview Blvd, Suite 310, A090 Aurora, CO 80045, USA; E-Mail: wsull@me.com; 5 Cincinnati Children’s Hospital Medical Center, 3333 Burnet Avenue, Cincinnati, OH 45229, USA; E-Mail: john.clancy@cchmc.org

**Keywords:** respiratory syncytial virus, matrix metalloproteinase, cell, murine model

## Abstract

Respiratory Syncytial Virus (RSV) is an important human pathogen associated with substantial morbidity and mortality. The present study tested the hypothesis that RSV infection would increase matrix metalloproteinase (MMP)-9 expression, and that MMP-9 inhibition would decrease RSV replication both *in vitro* and *in vivo*. RSV A2 infection of human bronchial epithelial cells increased MMP-9 mRNA and protein release. Cells transfected with siRNA against MMP-9 following RSV infection had lower viral titers. In RSV infected wild-type (WT) mice, MMP-9, airway resistance and viral load peaked at day 2 post infection, and remained elevated on days 4 and 7. RSV infected MMP-9 knockout (KO) mice had decreased lung inflammation. On days 2 and 4 post inoculation, the RSV burden was lower in the MMP-9 KO mice compared to WT controls. In conclusion, our studies demonstrate that RSV infection is a potent stimulus of MMP-9 expression both *in vitro* and *in vivo*. Reduction of MMP-9 (via siRNA knockdown, and in MMP-9 KO mice) resulted in decreased viral replication. Our findings suggest MMP-9 is a potential therapeutic target for RSV disease.

## 1. Introduction

Respiratory Syncytial Virus (RSV) infects virtually all infants worldwide by two years of age and is a major cause of acute respiratory failure in early childhood [1,2,3,4]. In addition, the occurrence of RSV bronchiolitis and pneumonia in premature infants, even in those without chronic lung disease, is associated with a significantly higher risk of hospitalization, intensive care unit admission, need for mechanical ventilation, and death [5,6,7,8,9,10]. Despite the substantial short and long-term morbidity, and mortality associated with pediatric RSV infection, management remains limited to supportive care. Passive immunization against RSV is currently available in the form of a monoclonal antibody (Palivizumab, MedImmune; [11]) but its administration does not always prevent development of disease, hospital admissions, or resource utilization [7,12,13]. The only approved antiviral treatment for RSV in children is inhaled ribavirin [14] but its use is associated with potential teratogenicity, and its efficacy is questioned [15].

Matrix metalloproteinase (MMP)-9 is a protease that has been implicated in the pathogenesis of several well-recognized pulmonary disorders, including cystic fibrosis, acute lung injury (ALI), and asthma [16,17,18]. We recently reported higher MMP-9 activity in lung secretions of intubated children with RSV bronchiolitis when compared to controls, and found a positive correlation between early elevation of MMP-9 and disease severity [19]. In the current study, we tested the hypothesis that RSV infection is sufficient to stimulate expression and release of MMP-9 from human airway epithelial cells, and enhance pulmonary MMP-9 release *in vivo*. Due to the direct positive relationship that we observed between MMP-9 levels and disease severity in our observational trial, we also tested the hypothesis that reduced MMP-9 levels or activity would suppress RSV replication *in vitro* and *in vivo*.

## 2. Results

### 2.1. RSV Infection of 16HBE Cells Led to an Increase in MMP-9 mRNA and Protein Release

**Figure 1:**
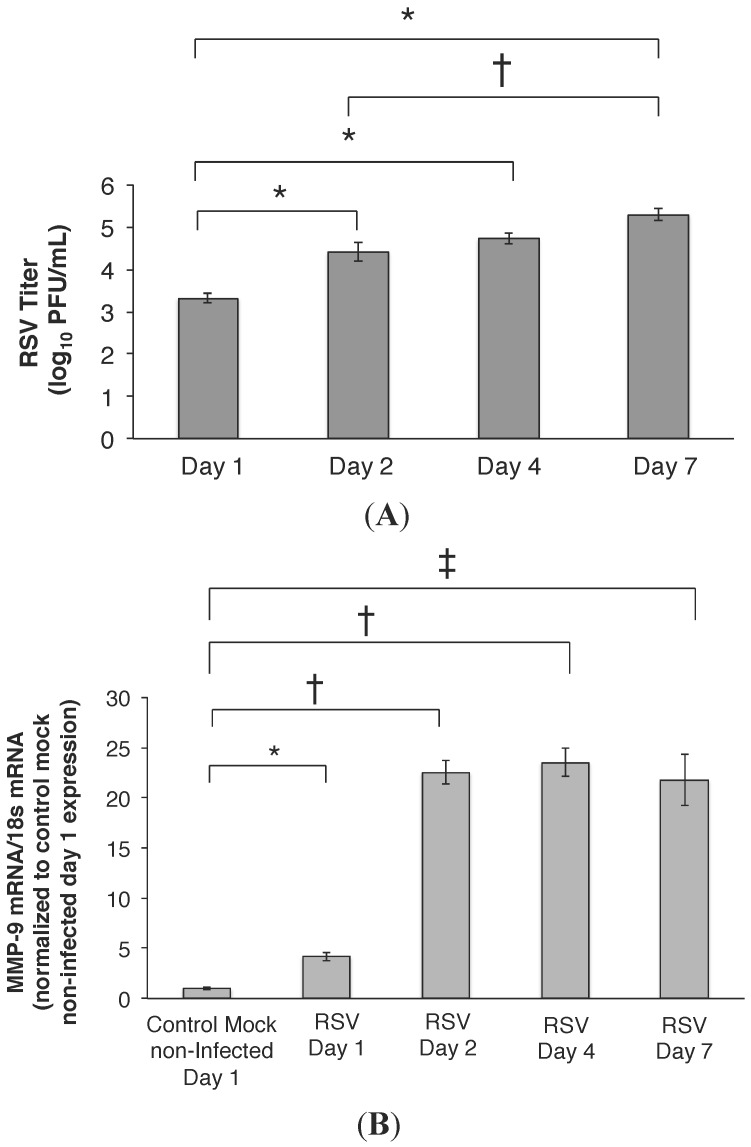

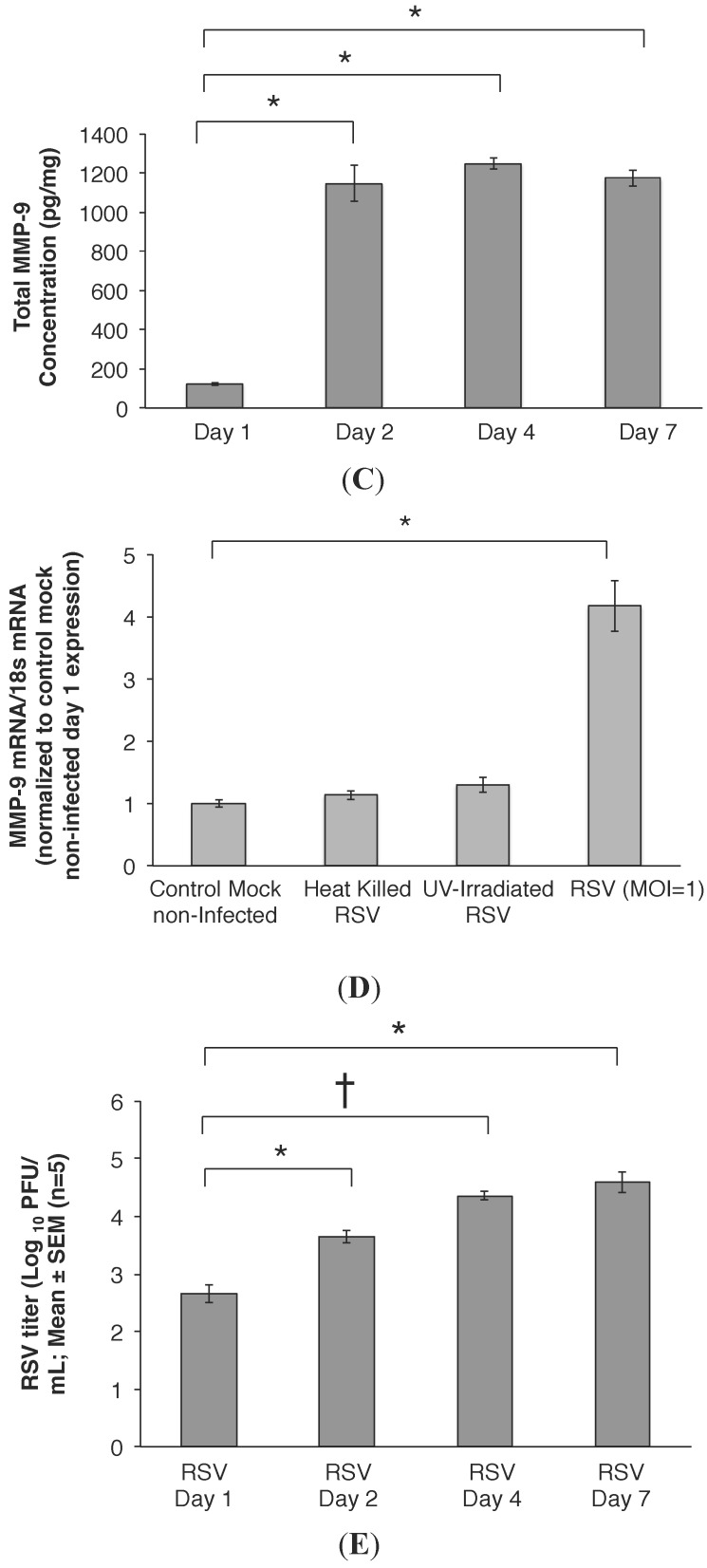

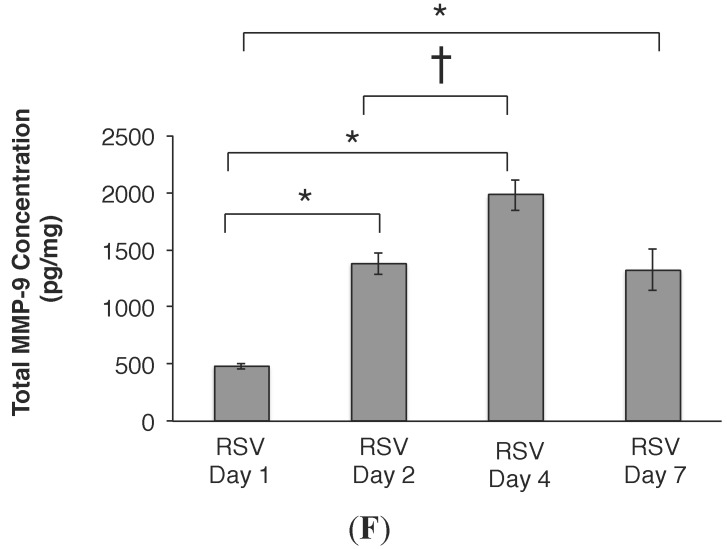
(**A**) RSV titer is increased in infected HBE cells over time. 16HBE cells were permissive to RSV infection, with increasing viral titer detected over time. At 24 h PI, the RSV titer was 3.2 ± 0.1 log_10_ PFU/mL, which increased to 4.4 ± 0.2 and 4.7 ± 0.1 log_10_ PFU/mL, respectively, on days 2 and 4 PI. By day 7 PI, the RSV titer was approximately 1–2 logs higher compared to the 24 h and 48 h time point. Data represent mean ± SEM. * *p* < 0.001 and ^†^ *p* = 0.003 as indicated, by ANOVA followed by Tukey-Kramer multiple comparisons test; (**B**) RSV infection is a potent stimulus of MMP-9 mRNA expression. MMP-9 mRNA was four-fold higher in the RSV infected HBE cells (MOI = 1) compared to control (mock non-infected) cells on day 1 (* *p* < 0.03). MMP-9 mRNA levels remained increased on days 2 to 7 post RSV infection, and were approximately 21–23-fold higher compared to control non-infected cells. Data represent mean ± SEM. ^†^ *p* < 0.01 and ^‡^ *p* < 0.002 as indicated, by ANOVA followed by Tukey-Kramer multiple comparisons test; (**C**) Total MMP-9 concentration is increased in RSV infected cell media. Total MMP-9 concentration in the media of RSV infected 16HBE cells first peaked on day 2 PI compared to day 1, correlating with the increased in MMP-9 mRNA measured (Figure 1B). MMP-9 concentration remained increased on day 4 and 7 PI, relative to levels measured on day 1. Data represent mean ± SEM. * *p* < 0.001 as indicated, by ANOVA followed by Tukey-Kramer multiple comparisons test; (**D**) Viral infectivity is necessary for induction of MMP-9 expression in human airway epithelial cells. Exposure of 16HBE cells to heat killed and UV light treated RSV (MOI = 1) failed to stimulate increases in MMP-9 mRNA, suggesting that MMP-9 gene expression and protein release was specific to RSV infection (and not just to viral structural protein exposure). MMP-9 mRNA was approximately four-fold higher in the RSV infected cells (MOI = 1) compared to control mock non-infected, heat killed and UV-irradiated RSV exposed cells. Data represent mean ± SEM. * *p* < 0.03; (**E**) Primary human lower airway cells are permissive to RSV infection. Primary human airway cells were obtained from lung transplant recipients, and grown at an air–liquid interface. Cells were infected with RSV at MOI of 1, and viral titers were determined over time. On day 1 PI, RSV titer was 2.7 ± 0.1 log_10_ PFU/mL, which increased to approximately 4–5 log_10_ PFU/mL by days 2 to 7 PI. Data represent mean ± SEM. * *p* < 0.01 and ^†^ *p* < 0.002 as indicated, by ANOVA followed by Tukey-Kramer multiple comparisons test; (**F**) RSV infection induces MMP-9 release from primary human airway epithelial cells. MMP-9 levels were detected consistently in the apical media of RSV infected primary airway cells. Levels on days 2 to 7 PI were higher than MMP-9 concentration measured on day 1, with peak concentration found on day 4 PI. Data represent mean ± SEM. * *p* < 0.0001 and ^†^ *p* < 0.001 as indicated, by ANOVA followed by Tukey-Kramer multiple comparisons test.

Airway epithelia were permissive to RSV infection with virus detected in the cell media by 24 h post-infection (PI) (3.3 ± 0.1 log_10_ PFU/mL, Figure 1A). At 48 h PI, viral load increased to 4.4 ± 0.2 (log_10_ PFU/mL; *p* < 0.001) and remained increased on day 7 PI (5.3 ± 0.1 log_10_ PFU/mL; *p* < 0.001). Transepithelial resistance (TER) was measured daily as a surrogate for monolayer integrity, and remained stable through day 7 PI (Day 1; 483 ± 10 Ω.cm^2^, Day 7; 825 ± 9 Ω.cm^2^; *p* < 0.0009). We next examined whether RSV was a stimulus for MMP-9 expression and release from the airway epithelia. At a MOI of 1, RSV infection led to a four-fold increase in MMP-9 mRNA transcription on day 1 PI compared to control mock non-infected cells, and MMP-9 mRNA remained elevated through day 7 PI (Figure 1B). Increased total MMP-9 protein was also measured in the media of RSV infected cells, following a similar time course (Figure 1C) and correlating with the increase in MMP-9 mRNA. Exposure of airway epithelium to heat killed and UV light treated virus failed to stimulate an increase in MMP-9 mRNA (Figure 1D), suggesting that MMP-9 gene expression was specific to replication-competent RSV infection (and not just to viral antigen). Figure 1E,F similarly show that primary human airway epithelial cells were permissive to RSV infection, and led to an increase in MMP-9 release, when compared to uninfected control cells. However, due to inconsistent MMP-9 knockdown, 16HBE cells were used for all the subsequent *in vitro* experiments.

### 2.2. MMP-9 Knockdown in 16HBE Cells Decreased RSV Titer

**Figure 2:**
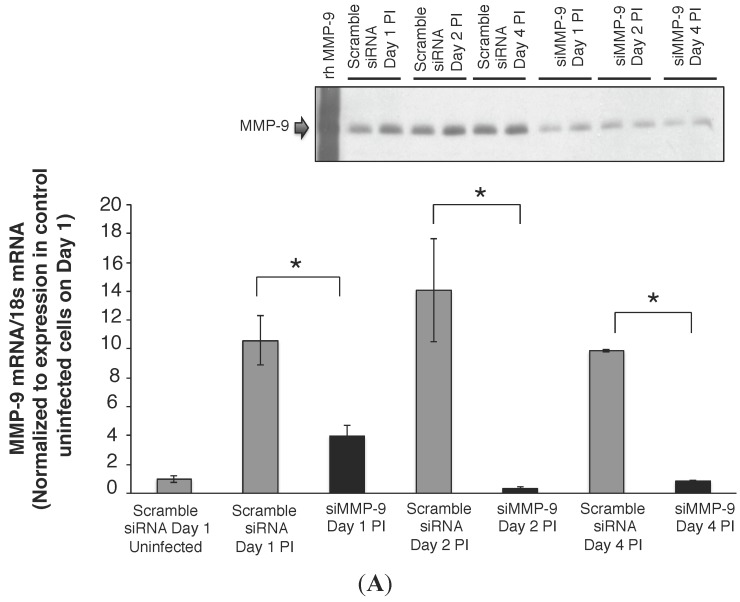

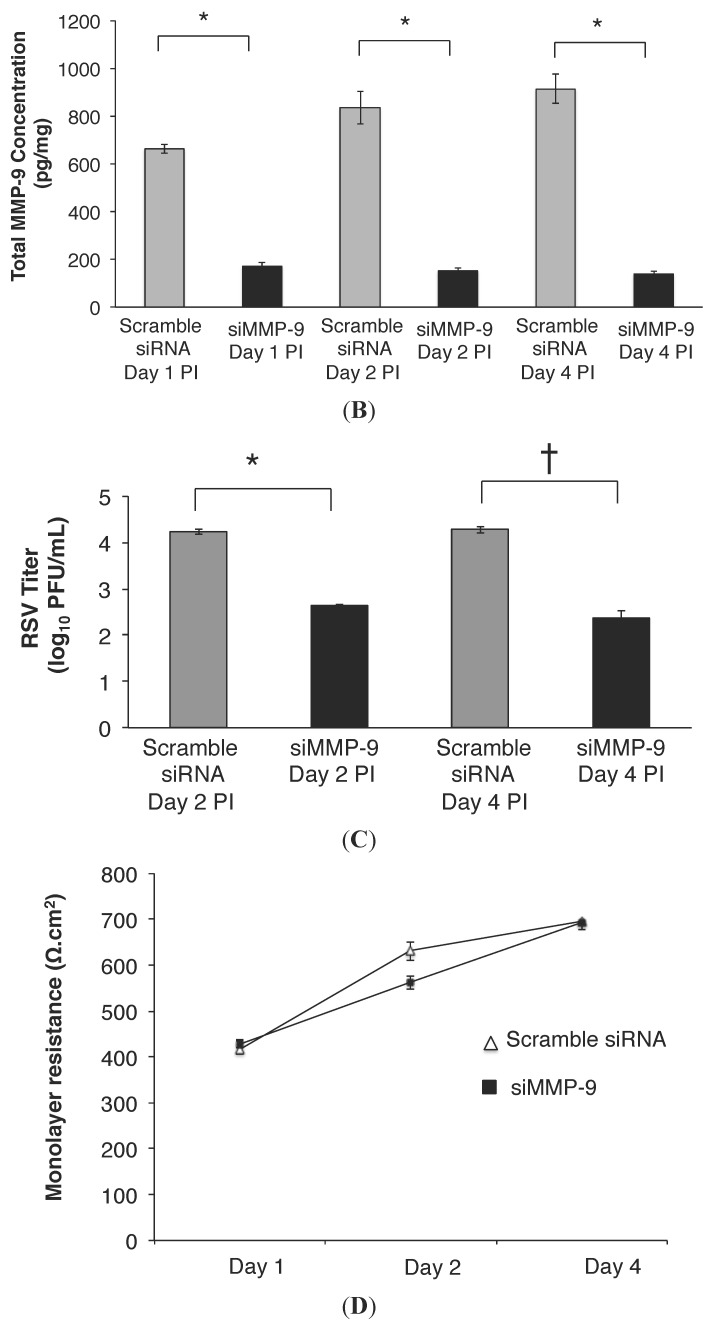
(**A**) MMP-9 knockdown in 16HBE cells using siRNA. RSV infection (MOI = 1) was performed 24 h post transfection with siRNA (s8864 at 10 μL; Ambion) against MMP-9. Transient transfection of siRNA s8864 resulted in knockdown of MMP-9 by 60%–80% on day1–4 PI. In contrast, cells transfected with scramble siRNA had increased MMP-9 transcription on day 1–4 PI compared to control uninfected cells, similar to our previous observation (Figure 1B). Data represent mean ± SEM. * *p* < 0.0001 as indicated, by ANOVA followed by Tukey-Kramer multiple comparisons test. MMP-9 protein (92 kDa) detected was less in the media obtained from the apical compartment of knockdown cells (siMMP-9), compared to cells transfected with scramble siRNA. Each lane represents a sample from a separate well (Figure inset); (**B**) Total MMP-9 release is decreased in 16HBE cells following MMP-9 Knockdown. Total MMP-9 concentration in the apical media of HBE cells transfected with scramble siRNA (control) *vs*. siRNA directed against MMP-9 (siMMP-9) were measured at days 1, 2, and 4 post RSV infection (MOI = 1). On Day 1 of MMP-9 knockdown, MMP-9 levels were decreased by 74% and remained decreased by greater than 80% on days 2 and 4 post infection, compared to controls. *n* = 5 wells per group of experiment, at each time point. Data represent mean ± SEM. * *p* < 0.00001 as indicated, by ANOVA followed by Tukey-Kramer multiple comparisons test; (**C**) RSV titer is lower in MMP-9 knockdown 16HBE cells. RSV titer was 1.6 logs lower in cells transfected with siRNA directed against MMP-9 when compared to control cells transfected with scramble siRNA (2.4 ± 0.02 log_10_ PFU/mL *vs*. 4.2 ± 0.06 log_10_ PFU/mL). At day 4 PI, the RSV titer remained lower in the MMP-9 knockdown cells compared to control cells (2.3 ± 0.1 log_10_ PFU/mL *vs*. 4.3 ± 0.08 log_10_ PFU/mL). Data represent mean ± SEM. * *p* < 0.00007 and ^†^ *p* < 0.0001 as indicated, by ANOVA followed by Tukey-Kramer multiple comparisons test; (**D**) Transepithelial resistance is unaffected by MMP-9 knockdown in 16HBE cells. Transepithelial resistance (TER) measurements were used as a surrogate for cell health and viability, as it requires retention of the tight junctional apparatus. We obtained TER measurements at each time point in the RSV infected HBE cells transfected with scramble siRNA (control, white triangle) and siRNA directed against MMP-9 (black square). TER measurements were similar between control and MMP-9 knockdown cells on day 1 PI. Although, the TER measurements were higher on day 2 PI in the scramble siRNA control cells, the TER measurements were similar on day 4 PI, indicating maintenance of monolayer integrity. Data represent mean ± SEM.

To further examine the relationship between MMP-9 and RSV infection, we tested whether MMP-9 expression contributed to RSV replication. siRNA knockdown of MMP-9 in RSV infected 16HBE cells decreased MMP-9 mRNA levels by greater than 60% on day 1, and greater than 80% on days 2 and 4 PI, compared to control infected cells (scramble siRNA, Figure 2A). Concomitant reductions in MMP-9 protein release (Figure 2A inset) and total MMP-9 concentrations (Figure 2B) were also observed in the cell media following knockdown compared to controls on days 1, 2, and 4 post RSV infection. MMP-9 knockdown reduced RSV titer by 1.6 logs compared to control conditions on day 2 PI (Figure 2C; *p* < 0.0001), and 1.8 logs on day 4 PI compared to scramble siRNA-treated cells (Figure 2C; *p* < 0.0001). To monitor monolayer integrity, we measured TER, and found the values to be similar between RSV infected monolayers transfected with siRNA against MMP-9, and controls transfected with scrambled siRNA (Figure 2D). The TER measurements also remained stable throughout the duration of the experiment (day 4 control scramble: 696 ± 18 Ω.cm^2^ *vs.* day 4 siRNA MMP-9: 692 ± 4 Ω.cm^2^), indicating retention of the tight junction apparatus.

### 2.3. RSV Infection Led to an Early Increase in MMP-9 and MMP-9:TIMP-1 Ratios in Vivo

**Figure 3:**
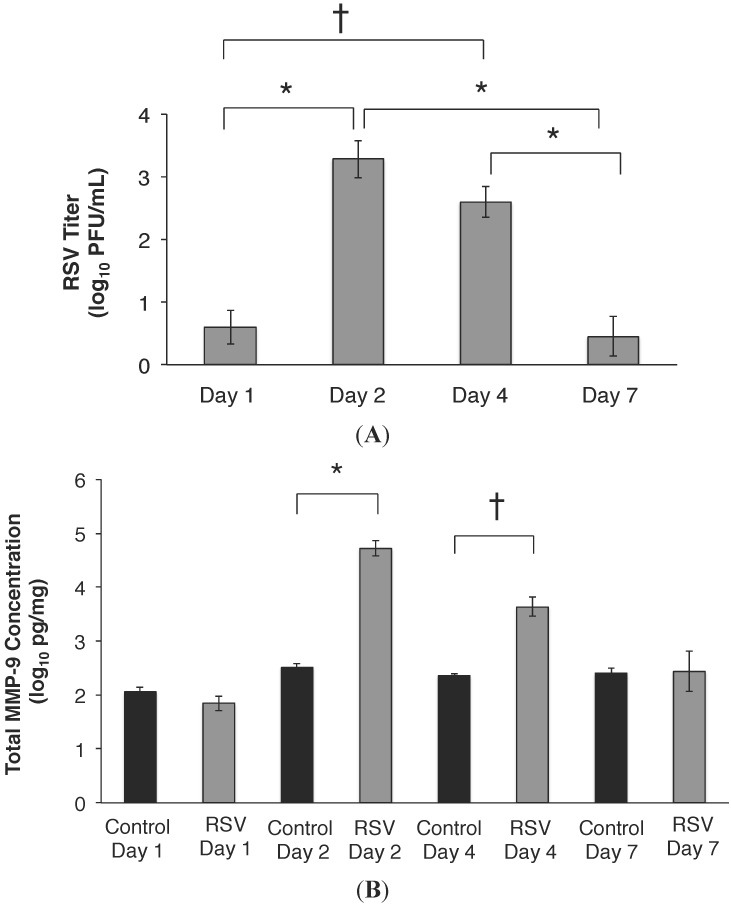

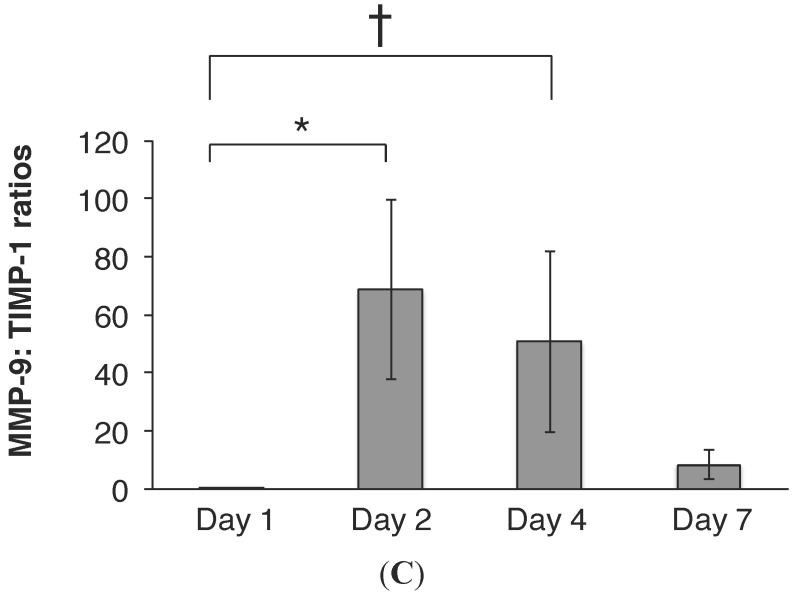
(**A**) RSV titer PI in wild-type (WT) C57BL/6 mice with time of infection. RSV was detected consistently in BALF by 24 h after inoculation. The highest viral titer was measured on day 2 PI (3.2 ± 0.3 log_10_ PFU/mL *vs*. 0.6 ± 0.2 log_10_ PFU/mL on Day 1), and remained increased on day 4 (2.6 ± 0.2 log_10_ PFU/mL). By day 7, the viral titer was decreased by approximately 3 logs (0.4 ± 0.3 log_10_ PFU/mL), and similar to that detected on day 1. *n* =20 mice; 5 mice per group x 4 separate experiments, for each time point. Data represent mean ± SEM. * *p* < 0.001 and ^†^ *p* = 0.004 as indicated, by ANOVA followed by Tukey-Kramer multiple comparisons test; (**B**) Total MMP-9 concentration peaks on day 2 post RSV inoculation. Total MMP-9 concentration measured in BALF of RSV infected WT mice peaked on day 2 PI, and was 2.2 logs higher compared to sham-infected controls. MMP-9 levels remained increased on day 4 PI, albeit lower than day 2 PI. By day 7 PI, similar MMP-9 concentrations were measured in RSV infected and control mice. Data represent mean ± SEM. * *p* < 0.0001 and ^†^ *p* < 0.005 as indicated, by ANOVA followed by Tukey-Kramer multiple comparisons test; (**C**) MMP-9:TIMP-1 ratios *in vivo* post RSV infection. TIMP-1 is a natural inhibitor for MMP-9 activity *in vivo*, and MMP-9:TIMP-1 ratio was determined at all time points post RSV infection. The highest MMP-9:TIMP-1 ratios were measured on days 2, and remained high on 4 PI, correlating with the increased MMP-9 concentrations measured in the lung (Figure 3B). MMP-9:TIMP-1 ratio was decreased by greater than 50% by day 7 PI. Data represent mean ± SEM. * *p* = 0.005 and ^†^ *p* < 0.02 as indicated, by ANOVA followed by Tukey-Kramer multiple comparisons test.

C57BL/6 mice were infected with RSV as described in Methods (intranasal, 10^7^ PFU) and then studied by pulmonary function testing and bronchoalveolar lavage fluid (BALF) up to seven days PI. In the WT infected animals, the highest RSV titer was measured on days 2 and 4 PI, with decreasing titers measured on day 7 PI (Figure 3A). By day 14, and 21, the RSV titer in BALF was below the limit of detection (data not shown). The BALF MMP-9 concentration was similar between the RSV and control sham infected mice on days 1 and 7 PI (Figure 3B). On days 2, and 4 PI, MMP-9 concentration was approximately 1–2 logs higher compared to levels measured in the control sham infected animals (*p* < 0.0001 and 0.005; respectively). In RSV infected mice, total MMP-9 peaked on day 2 PI, in contrast to similar MMP-9 concentrations measured across time in the control group. The highest MMP-9:TIMP-1 ratios were similarly measured in RSV infected mice on days 2 and 4 PI (Figure 3C).

### 2.4. MMP-9 Knockout Mice have Decreased Inflammation and Viral Load during Acute RSV Infection

**Figure 4:**
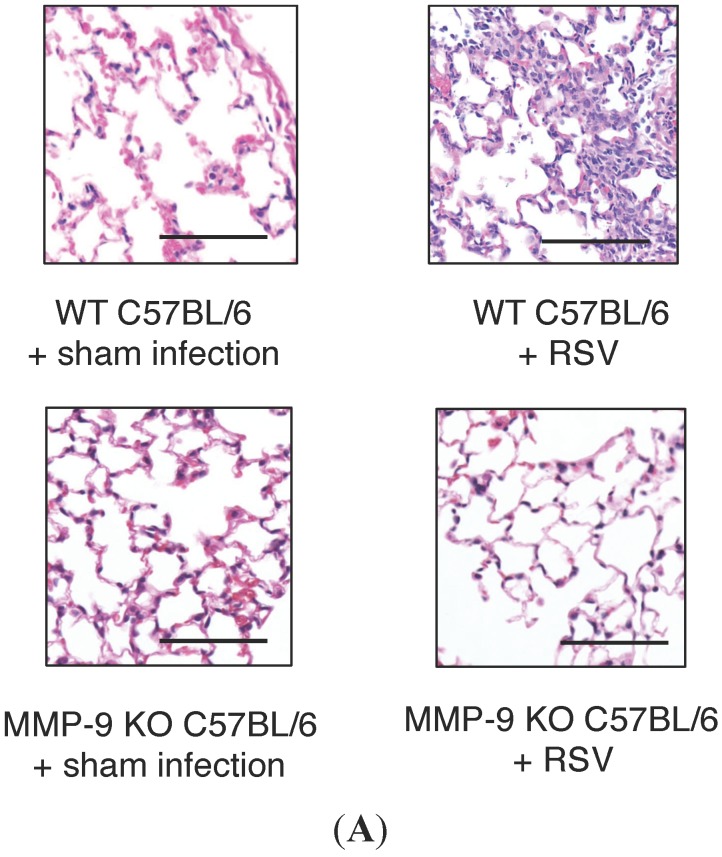

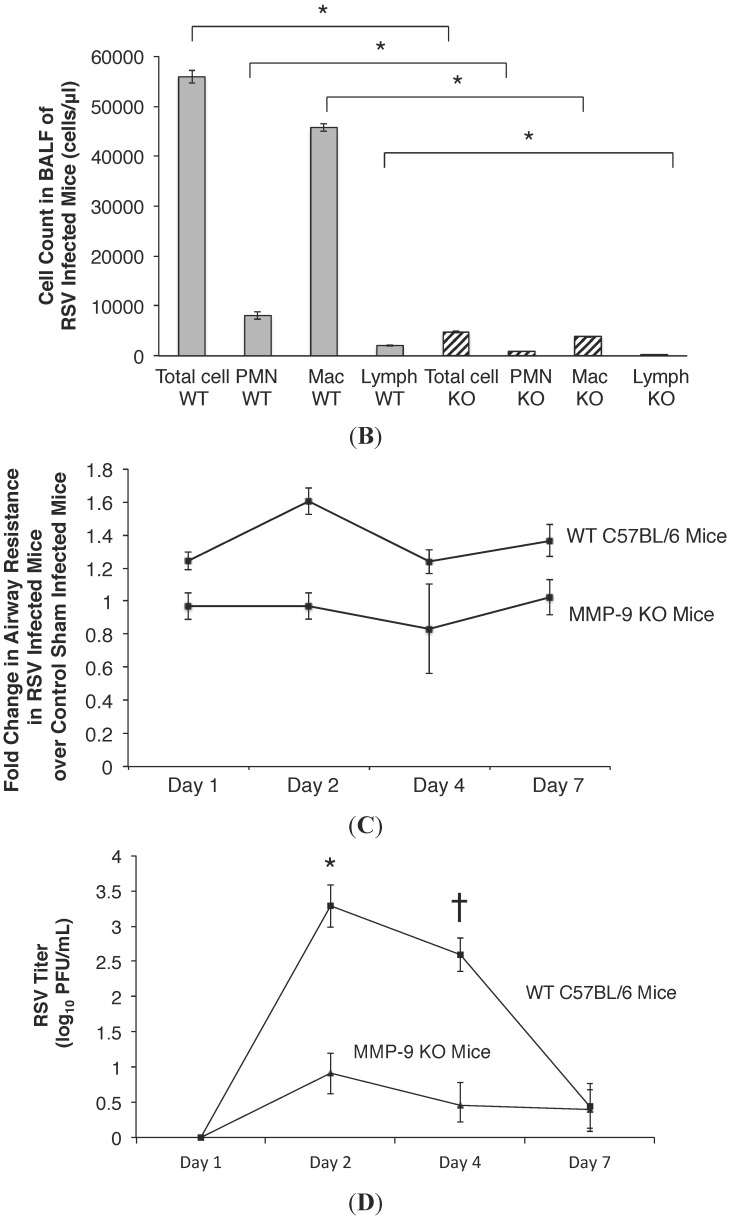
(**A**) Lung inflammation in RSV infected WT and MMP-9 KO mice. WT and MMP-9 KO C57BL/6 mice received the same quantity of RSV inoculum. Tissue was stained with hematoxylin and eosin to assess lung inflammation post RSV infection. Acute pneumonitis with perivascular and peribronchial inflammatory infiltrates was observed in the RSV infected WT animals on days 2 PI (*vs*. normal lung histology in mock infected WT controls). In contrast, minimal lung inflammation was noted in the RSV infected MMP-9 KO mice. A similar histologic pattern was observed in RSV infected and sham mock infected MMP-9 KO mice. Scale bar: 67 μm; (**B**) Decreased cellularity in BALF following RSV infection in MMP-9 KO mice relative to WT mice. Total cell count in BALF of RSV infected MMP-9 KO mice (*n* = 5; stripe column) was almost 11-fold lower than RSV infected WT C57BL/6 mice (*n* = 5; grey column) on day 2 PI. Approximately 80% of cells found in both MMP-9 KO and WT mice were macrophages. The absolute macrophage count in the MMP-9 KO mice was 3814 *vs*. 45,737 cells/μL in the WT mice, respectively. In the MMP-9 KO mice, 19% of the total cell count was neutrophils (897 cells/μL), with lymphocytes accounting for less than 1% of cells recovered. In the WT mice, 14% of the total cell count was neutrophils (8060 cells/μL), and 4% was lymphocytes. Data represent mean ± SEM. * *p* < 0.04 as indicated, by ANOVA followed by Tukey-Kramer multiple comparisons test. PMN = neutrophils; Mac = macrophages; Lymph = lymphocytes; (**C**) Airway resistance is elevated in RSV infected WT mice compared to sham infected WT controls, but remains minimally changed following RSV infection in MMP-9 KO mice. WT and MMP-9 KO C57BL/6 mice (*n* = 10 per time point) were inoculated intranasally with10^7^ PFU of live RSV, and airway resistance was measured from the time of inoculation to day 7 PI. Airway resistance was similar between MMP-9 KO mice inoculated with RSV *vs*. those given a sham infection throughout the duration of infection. This is in contrast to increased airway resistance measured in RSV infected WT mice over time of infection when compared to control sham infected mice. On day 1 PI, airway resistance was 1.24 fold higher in RSV infected WT mice compared to control mice. Peak airway resistance in the RSV WT mice was measured on day 2 PI, with a 1.6 fold increase in resistance measured compared to sham infected controls. Airway resistance remained increased on days 4 and 7 PI in the WT mice. Data represent mean ± SEM; (**D**) RSV titer in WT and MMP-9 KO mice following RSV infection. WT and MMP-9 KO C57BL/6 mice were inoculated with 10^7^ PFU/mL of RSV, and viral titer was determined in BALF over multiple time points post infection. The RSV titer was approximately 2 logs lower in MMP-9 KO mice compared to WT mice on days 2 and 4 post infection. The viral burden was similar between MMP-9 KO and WT mice on day 1 and 7. Data represent mean ± SEM. * *p* < 0.001 and ^†^ *p* < 0.002 as indicated, by ANOVA followed by Tukey-Kramer multiple comparisons test.

To test the hypothesis that MMP-9 plays a critical role in RSV infection *in vivo*, MMP-9 KO C57BL/6 mice were infected with RSV (intranasal, 10^7^ PFU) as described previously, and lung inflammation, pulmonary function and viral load was determined PI. Figure 4A demonstrates that RSV infected MMP-9 KO mice had minimal lung inflammation, with no difference in lung histology noted between RSV infected and control sham infected MMP-9 KO mice. This is in contrast to WT mice, in which RSV infection led to the development of acute pneumonitis and an increase in lung cellular infiltrates (*vs*. control sham infected WT animals). The BALF cell count was also measured and found to be 11-fold higher in the RSV infected WT mice compared to RSV infected MMP-9 KO mice (Figure 4B, *p* < 0.04). The observed BALF cell types in both the RSV infected WT and MMP-9 KO mice were predominantly macrophages, followed by neutrophils and lymphocytes. At baseline, prior to RSV infection, airway resistance in the MMP-9 KO mice was greater than WT mice (Day 1 control MMP-9 KO mice: 0.9 ± 0.08 cm H_2_O.s/mL *vs.* 0.55 ± 0.04 cm H_2_O.s/mL in control WT mice; *p* < 0.002). However, in MMP-9 KO mice infected with RSV, airway resistance was similar to uninfected MMP-9 KO controls at all time points PI (Figure 4C). This is in contrast to greater airway resistance measured in the RSV infected WT mice compared to sham infected WT controls on days 1–7 PI, with the highest resistance measured on day 2 PI (1.1 ± 0.08 cm H_2_O.s/mL in WT RSV mice *vs.* 0.66 ± 0.03 cm H_2_O.s/mL in WT controls; *p* < 0.002). On days 4 and 7 PI, airway resistance remained increased in the RSV infected WT mice compared to sham infected controls (0.83 ± 0.07 cm H_2_O.s/mL in WT RSV mice *vs.* 0.67 ± 0.01 cm H_2_O.s/mL in WT controls on day 4 PI; *p* < 0.04 and 0.89 ± 0.09 cm H_2_O.s/mL in WT RSV mice *vs.* 0.66 ± 0.06 cm H_2_O.s/mL in WT controls on day 7 PI; *p* = 0.07; respectively). The pulmonary RSV titer was also measured over multiple time points post infection by plaque assay of lung tissue homogenates. We found that the RSV titer was reduced by 2–3 logs in the MMP-9 KO mice relative to WT controls on days 2 and 4 PI (Figure 4D). By day 7, RSV titer was similar between the WT and MMP-9 KO groups. A positive correlation was found between BALF MMP-9 concentrations and RSV titers on days 2, 4, and 7-post infection in the WT mice (Table 1). In contrast, no correlation was seen between TIMP-1 and RSV titer.

**Table 1:** Positive correlation between MMP-9 levels and RSV titer in wild-type C57BL/6 mice.

	r	p
MMP-9 and RSV Day 2 Post Infection	0.66	0.002
TIMP-1 and RSV Day 2 Post Infection	0.37	0.11
MMP-9:TIMP-1 and RSV Day 2 Post Infection	0.13	0.58
MMP-9 and RSV Day 4 Post Infection	0.46	0.042
TIMP-1 and RSV Day 4 Post Infection	0.05	0.83
MMP-9:TIMP-1 and RSV Day 4 Post Infection	0.44	0.04
MMP-9 and RSV Day 7 Post Infection	0.64	0.04
TIMP-1 and RSV Day 7 Post Infection	0.09	0.83
MMP-9:TIMP-1 and RSV Day 7 Post Infection	0.66	0.07

## 3. Discussion

The current management of pediatric RSV infection consists of supportive care, including oxygen supplement, adequate hydration, and mechanical ventilation for those who develop respiratory failure. Multiple therapeutic strategies have been explored with very limited success [20,21,22], and a vital need remains for effective therapy. Recently, DeVincenzo *et al.* demonstrated that treatment with an RSV entry inhibitor reduced the viral load and the severity of clinical disease in a challenge study of healthy adults, but its efficacy is untested in natural infection, especially in infants and children [23].

In the current study, we examined relationships between RSV infection and MMP-9 expression using *in vitro* and *in vivo* model systems. We demonstrated that RSV infection was a potent stimulus of MMP-9 expression and release from human airway epithelial cells, that reduced MMP-9 expression resulted in decreased viral titer, and that these observations extended to an *in vivo* mouse model of RSV bronchiolitis. In addition to lower viral burden, lung inflammation and airway resistance were also attenuated in MMP-9 KO mice compared to WT controls during acute infection. To the best of our knowledge, this is the first study to demonstrate these positive relationships between RSV infection and MMP-9, and decreased viral burden in conditions lacking MMP-9 *in vitro* and *in vivo.*

MMP-9 is a member of the matrix metalloproteinase family of zinc and calcium dependent endopeptidases. Recent studies have implicated MMP-9 dysregulation to be associated with several lung disorders, such as chronic obstructive lung disease (COPD), ALI, asthma and chronic lung disease of prematurity [24,25,26,27]. Disruption of the balance between MMP-9 and endogenous inhibitors (including TIMP-1, also described as protease/antiprotease imbalance) has been implicated in the pathology of these pulmonary diseases. We recently reported significantly higher MMP-9 activity in lung secretions of pediatric subjects who had respiratory failure secondary to RSV infection compared to those who had non-RSV ALI [19], and a positive association between early MMP-9 elevation and disease severity. This relationship was specific for MMP-9 activity, as neither human neutrophil elastase nor myeloperoxidase activity predicted subsequent lung disease severity in either the RSV or control groups. Furthermore, excess MMP-9 activity might be a logical target to treat the aforementioned diseases (such as COPD and asthma) [28,29].

Because of the direct relationship between MMP-9 and RSV disease, coupled with its potential as a therapeutic target, we tested the impact of MMP-9 expression first in an *in vitro*, then *in vivo* animal model of RSV bronchiolitis. In our human bronchial epithelial cell model, we demonstrated that the RSV titer was reduced in cells following MMP-9 knockdown, and that this inhibitory effect was observed over several days PI. The results suggest a positive and permissive role for MMP-9 in RSV replication and/or release, a conclusion that is supported by our *in vivo* studies. Using a single gene knockout strategy in our *in vivo* model, we found that RSV titer was reduced greater than 2 logs in the MMP-9 knockout mice during the acute phase of infection (day 2–4). Furthermore, the airway resistance of MMP-9 knockout mice infected with RSV was similar to non-infected MMP-9 knockout controls, which contrasted the increased resistance caused by RSV infection in MMP-9 expressing mice. Lung inflammation was also found to be minimal in RSV infected MMP-9 KO mice, having a similar histologic appearance as control sham infected MMP-9 KO mice. This is in contrast to the observed development of acute pneumonitis in RSV infected WT animals. Consistent with the histologic observations, significantly fewer neutrophils, macrophages and lymphocytes in the bronchoalveolar lavage fluid were also detected in RSV infected MMP-9 KO as compared to WT mice.

Others have demonstrated that excessive pulmonary MMP-9 activity can contribute to increased mortality during infection, with improved survival demonstrated in MMP-9 knockout mice compared to MMP-9+ littermates with respiratory tularemia [30]. MMP-9 inhibition has also been demonstrated to reverse smoke-induced airspace enlargement [31] and attenuate lung inflammation in ALI animal models [32,33]. In a study by Cataldo *et al.*, reduced airway inflammation was observed in MMP-9 KO animals sensitized and exposed to allergens, compared to WT controls [34]. In this murine model of asthma, the investigators found that airway resistance of MMP-9 KO mice was not different after exposure to ovalbumin, and these mice failed to show significant airway hyperresponsiveness to carbachol, as compared to WT mice. In a model of ALI induced by immune complexes, Warner *et al.* found that MMP-9 KO mice displayed less severe lung injury than WT controls [35]. MMP-9 deficient mice were also shown to be protected from obliterative bronchiolitis as evidenced by reduced neutrophil influx and collagen deposition [36]. In the current study, we have shown that MMP-9 is important in RSV disease, as absence of MMP-9 *in vivo* led to decreased lung inflammation, viral burden, and attenuated the increase in airway resistance produced by acute RSV infection.

This study has several limitations. First, RSV infection in mice is different than human infection. WT mice are described as semi-permissive hosts for human RSV. In the most permissive mouse strains, such as BALB/c, a very high intranasal inoculum (up to 10^7^ PFU per mouse) is required to detect lower respiratory tract symptoms and general signs of illness [37,38]. In our studies, we found that C57BL/6 mice were also permissive to RSV infection, as supported by others who have shown that RSV-induced inflammation is independent of murine genetic background [39]. We similarly used a high viral inoculum, and still demonstrated an increase in viral titers over days 1–4, along with increases in lung inflammation and airway resistance. This is similar to findings reported by Chávez-Bueno *et al.* [39] who found peak RSV titer on days 3–5 PI in C57BL/6 mice, suggesting active viral replication. Furthermore, the use of this mice strain mirrors the extensive experience with gene targeting against MMP-9 and, most importantly, the commercial availability of MMP-9 KO mice. Others have also used Sendai virus or PVM, a pneumovirus pathogen originally isolated from mouse lung tissue [40], which undergoes robust viral replication in the lower airways in response to a minimal virus inoculum (200 PFU) [41,42], and are considered true infectious murine models of RSV. However, we chose not to use the PVM mouse model because of the difference in antigenicity between PVM and human RSV. Recent evidence also suggests that viral-host response and airway mechanics in mice may differ depending on the RSV strain used [43,44]. In this study, we focused on the role of MMP-9 in RSV infection based on our prior clinical observations, and did not include other critical host contributors to RSV pneumonia and respiratory failure. While the use of human RSV in C57BL/6 mice differed from other established infectious models, our findings confirm that RSV-induced elevation in MMP-9 contributes to disease pathogenesis. Future studies utilizing different strains of mice or virus will be important to further elucidate the host-viral response in RSV disease.

Taken together, our results show for the first time that there is a direct and positive feedback loop connecting RSV infection and MMP-9 expression. How RSV induces MMP-9 expression is still not fully elucidated, but work by others has shown that RSV stimulates a number of transcription factors including nuclear factor (NF)-κB and AP-1, which have been implicated in MMP-9 regulation [45,46]. How MMP-9 facilitates RSV infection is also unknown, but potential protein targets that are important to RSV replication and syncytia formation include the attachment and fusion protein. While these important questions are beyond the scope of the current manuscript, these pathways represent additional targets that are likely relevant to our comprehensive understanding of RSV infection and disease modification.

## 4. Materials and Methods

### 4.1. Cell Culture

Sixteen human bronchial epithelial (HBE) cells, a differentiated SV-40 transformed bronchial epithelial cell line [47], were a generous gift from Dr. D.C. Gruenert (University of California, San Francisco, CA, USA). For the *in vitro* experiments described, cells were seeded on cell culture inserts (Transwell permeable supports, diameter 12 mm, 0.4 μm pores; Corning, Acton, MA, USA) in multi-well plates and maintained under liquid-liquid conditions until they were confluent. Subsequently, apical medium was removed from the upper compartment to create an air-liquid interface. 16 HBE cells were maintained in minimum essential media (MEM) supplemented with 10% fetal bovine serum (FBS), nonessential amino acids, and penicillin-streptomycin (PS, Invitrogen, Carlsbad, CA, USA) in a 5% CO2-95% air incubator at 37 °C.

### 4.2. RSV Infection of Cell Model System

The RSV strain A2 was obtained from American Type Culture Collection (ATCC, Manassas, VA, USA) and cultured in HEp-2 human nasopharyngeal carcinoma cells (CCL-23, ATCC) using a previously described sucrose purification method [48]. RSV titers were determined by a plaque assay technique in HEp2 cells [49] and expressed as Plaque Forming Unit (PFU)/millimeter. 16 HBE cells were infected with RSV at an MOI = 1, and cell media/lysate were collected at days 1, 2, 4, and 7 post infection. To determine whether viral infectivity was required for MMP-9 stimulation, control experiments utilized heat-killed RSV (viral infectivity is eliminated and protein structures are changed) and UV-irradiated RSV (viral infectivity is eliminated without altering the conformation of viral proteins). For UV-inactivation of RSV, aliquots of RSV stocks were exposure to 1800 mJ of radiation in a Stratalinker UV cross-linker (Stratagene), and for heat-killed RSV, aliquots of viral stocks were boiled for 45 min [50].

### 4.3. Transepithelial Resistance Measurement

Transepithelial resistance (TER) provides a physical measure of the electrical resistance between airway epithelial cells and was monitored as a surrogate for monolayer integrity [51,52]. TER was measured daily using a World Precision Instruments ohmmeter (WPI, Inc., FL, USA), and results are reported as mean ± SEM Ω.cm^2^. Cell monolayers had resistance values of approximately 400 Ω.cm^2^ prior to RSV infection.

### 4.4. Real Time RT-PCR to Quantify MMP-9 mRNA Expression

TaqMan One Step RT-PCR protocol (Applied Biosystems, Foster City, CA, USA) was used to quantify MMP-9 mRNA transcripts using ‘Assays on Demand’ Gene Expression Products, coupled with the ABI Prism 7500 sequence detection system (Applied Biosystems, Grand Island, NY, USA), as previously described [53]. Briefly, total RNA was isolated using the Qiagen RNeasy mini kit, according to the manufacturer’s instructions (Qiagen, Valencia, CA, USA). To prevent possible DNA contamination, the samples were pretreated with RNase-free DNase (Qiagen). Sequence specific primers (20 μM) and probes (5 μM) for human MMP-9 and 18S rRNA were purchased from Assays on Demand (ABI, Grand Island, NY, USA). TaqMan One Step PCR Master Mix Reagents Kit (ABI) was used for reverse transcriptase and PCR. All experiments were run in triplicate on two separate days.

### 4.5. MMP-9 Knockdown in HBE Cells

Transient transfection of siRNA was performed using Lipofectamine^®^ 2000 Transfection Reagent (#11668, Life Technologies, New York, NY, USA) according to the manufacturer's instructions. 16HBE cells were seeded into a 6-well plate. siRNA (s8864; GACCTGGGCAGATTCCAAA; #4392420, Ambion, New York, NY, USA) at 10 μL was mixed with the transfection reagent (5 μL), and added to the cells. After transfection, cells were infected with RSV (MOI = 1) and cell media and lysate were collected on days 1, 2, and 4 PI, and stored at -80 until analysis.

### 4.6. Detection of MMP-9 by Western Blot Analysis

Media from the apical compartment were collected and electrophoresed on SDS-polyacrylamide gels and electroblotted onto Immobilon-P PVDF membranes. Membranes were blocked in Tris buffer (pH = 7.4) containing 5% powdered milk for one hour, washed, and incubated with primary MMP-9 antibody (MAB 911; R/D Systems) overnight at 4 °C. After incubation, samples were washed with borate saline (100 mM boric acid, 25 mM Na borate, 75 mM NaCl) and incubated with species-specific IgG horseradish peroxidase conjugates (at dilutions of 1:5000) for one hour. Immunoblots were then developed using ECL chemiluminescent kits (GE Healthcare, Piscataway, NJ, USA). 50 ng of recombinant MMP-9 (R/D Systems; Catalog #WBC018) was used as positive control. As a loading control, 50 μg protein/ sample was loaded for each immunoblot lane.

### 4.7. Measurement of MMP-9 and TIMP

Human and murine MMP-9 and TIMP concentration were quantified using an established ELISA assay (#DMP900; human MMP-9, #MMPT90; mouse MMP-9, #DTM 100; human TIMP, #MTM100; mouse TIMP; R&D Systems) following the manufacturer’s protocol. MMP-9 and TIMP concentrations (in pg) were adjusted for the amount of protein (in mg; Catalog #5000112; BioRad) that was present in the sample (reported as pg/mg).

### 4.8. Mice

Wild-type (WT) and congenic MMP-9 −/− C57BL/6 mice, 6–8 weeks old from Jackson Laboratories were maintained in filter-top cages and monitored daily for signs of respiratory distress. Food and water were provided ad libitum. All animal experiments were performed in animal biosafety level 2 facilities at the University of Alabama at Birmingham and were approved by Institutional Animal Care and Use Committee (#140809189) and in accordance with federal guidelines.

### 4.9. Inoculation

Mice were lightly anesthetized with inhaled isofluorane before intranasal inoculation with 10^7^ PFU of live RSV in 100 μL (2 aliquots of 50 μL each) of 10% MEM, as previously published [54]. Control mock-infected animals received an equal volume of supernatant from uninfected Hep-2 cells. For all studies, data for each experimental group were derived from a minimum of two independent infections.

### 4.10. Sample Collection

After measurement of lung function (see below), mice were euthanized and BAL specimens were obtained immediately to measure cytokine concentrations. Cell count in BALF was measured using a hemocytometer. In brief, cell suspensions were mixed with trypan blue solution, and with a cover-slip in place, a small amount of the trypan blue-cell suspension was placed into a chamber on the hemocytometer for counting. Histologic evaluations were performed on whole lung specimens (separate sets that had not undergone BAL) fixed with a 10% buffered formalin solution, embedded in paraffin, and stained with hematoxylin-eosin. Whole lung specimens were collected from another group of mice for quantification of viral loads by plaque assay. Specimens were obtained on days 1, 2, 4, and 7 PI.

### 4.11. Lung Function Analyses

Pulmonary function was measured in wild-type and congenic MMP-9−/− C57B/6 mice at days 1, 2, 4, and 7 PI. The flexiVent apparatus (SCIREQ, Montreal, QC, Canada) was used to perform measurements, including perturbations (predefined pressure of volume waveforms) including forced oscillations, via the tracheostomy using room air in the closed-chest animal [55]. For baseline, the delivered tidal volume was approximately 6 mL/kg, with a respiratory rate of 150/min. Measurements made included lung dynamic compliance and airway resistance (Rn or Raw; Newtonian resistance, which is primarily the resistance of the central or upper airways). Calibration of the flexiVent was done using the tracheal cannula to be used before each experiment. Mice were euthanized at the completion of the procedure.

### 4.12. Statistical Analysis

Descriptive statistics were computed for each study variable, including mean, standard error of the mean, median, and range. Since the distributions of the data for RSV infected animals, the BAL inflammatory markers (total MMP-9, TIMP-1, and MMP-9/TIMP-1 ratio), and two other continuous variables (resistance and compliance) deviated from a normal distribution, these data were logarithmically transformed using a log10 scale prior to statistical analysis. The log-transformed variables were determined to follow a normal or approximate normal distribution through the use of stem-and-leaf plots, normal probability plots and the Kolmogorov–Smirnov test. Comparisons between means were performed using analysis of variance (ANOVA). The Tukey–Kramer multiple comparisons test was used as the *post hoc* test of choice. Pearson correlation analysis was used to determine correlations between pairs of variables. Where possible, nonparametric statistical analyses (including the Kruskal–Wallis test and Spearman correlation analyses) corresponding to the above analyses were performed, and these analyses yielded results that are similar to those obtained by the parametric analyses described earlier. Statistical tests were two-sided and were performed using a 5% significance level (*i.e*., α = 0.05). Statistical analyses were performed using SAS software (version 9.4; SAS Institute, Inc., Cary, NC, USA).

## 5. Conclusions

In summary, we have demonstrated that RSV is a potent stimulus for MMP-9 release *in vitro* and *in vivo*. Reduction of MMP-9 (via siRNA knockdown, and in MMP-9 KO mice) resulted in decreased viral titers. In addition to decreased viral burden, lung inflammation and airway resistance was also attenuated in MMP-9 knockout mice compared to MMP-9 competent controls during the acute phase of infection. Together, the results demonstrate the antiviral and anti-inflammatory effect of MMP-9 inhibition in RSV infection. Our study suggests that further clinical studies are necessary to further elucidate the potential role of MMP-9 inhibition in human RSV disease, and to explore the mechanistic relationships between MMP-9 and RSV biology.
