# *Addendum*: Kong, M.Y.; Whitley, R.J.; Peng, N.; Oster, R.; Schoeb, T.R.; Sullender, W.; Ambalavanan, N.; Clancy, J.P.; Gaggar, A.; Blalock J.E. Matrix Metalloproteinase-9 Mediates RSV Infection *in Vitro* and *in Vivo*. *Viruses* 2015, *30*, 7, 4230–4253

**DOI:** 10.3390/v7102889

**Published:** 2015-10-26

**Authors:** Michele Y. F. Kong, Richard J. Whitley, Ning Peng, Robert Oster, Trenton R. Schoeb, Wayne Sullender, Namasivayam Ambalavanan, John Paul Clancy, Amit Gaggar, J. Edwin Blalock

**Affiliations:** 1 Departments of Pediatrics, University of Alabama at Birmingham, PPS 102, 1600 5th Ave South, Birmingham, AL 35233, USA; rwhitley@peds.uab.edu (R.J.W.); npeng@peds.uab.edu (N.P.); nambalavanan@peds.uab.edu (N.A.); 2 Departments of Medicine, University of Alabama at Birmingham, PPS 102, 1600 5th Ave South, Birmingham, AL 35233, USA; roster@uabmc.edu (R.O.); agaggar1@uab.edu (A.G.); blalock@uab.edu (J.E.B.); 3 Departments of Genetics, University of Alabama at Birmingham, PPS 102, 1600 5th Ave South, Birmingham, AL 35233, USA; trs@uab.edu; 4 Center for Global Health, Colorado School of Public Health, 13199 E Montview Blvd, Suite 310, A090 Aurora, CO 80045, USA; wsull@me.com; 5 Cincinnati Children’s Hospital Medical Center, 3333 Burnet Avenue, Cincinnati, OH 45229, USA; john.clancy@cchmc.org

The authors wish to make the following addition to their paper [1]. Gaggar, Amit and Blalock, J. Edwin contributed equally to this work. An Acknowledgement section was added and should read:

## References

[1] Kong M.Y., Whitley R.J., Peng N., Oster R., Schoeb T.R., Sullender W., Ambalavanan N., Clancy J.P., Gaggar A., Blalock J.E. (2015). Matrix Metalloproteinase-9 Mediates RSV Infection *in Vitro* and *in Vivo*. Viruses.

